# Context-Aware Sentence Classification of Radiology Reports Using Synthetic Data: Development and Validation Study

**DOI:** 10.2196/86365

**Published:** 2026-04-10

**Authors:** Tomohiro Kikuchi, Yosuke Yamagishi, Kohei Yamamoto, Toshiaki Akashi, Harushi Mori, Hisaki Makimoto, Takahide Kohro

**Affiliations:** 1 Data Science Center, Jichi Medical University, Tochigi, Japan; 2 Department of Radiology, Jichi Medical University, 3311-1, Yakushiji, Shimotsuke, Tochigi, 329-0498, Japan, 81 285-58-7362; 3 Department of Computational Diagnostic Radiology and Preventive Medicine, The University of Tokyo Hospital, Tokyo, Japan; 4 Division of Radiology and Biomedical Engineering, Graduate School of Medicine, The University of Tokyo, Tokyo, Japan; 5 Department of Radiology, Juntendo University School of Medicine, Tokyo, Japan

**Keywords:** natural language processing, artificial intelligence, large language models, data annotation, radiology report

## Abstract

**Background:**

Automated structuring of radiology reports is essential for data utilization and the development of medical artificial intelligence models. However, manual annotation by experts is labor-intensive, and processing real clinical data through commercial large language models (LLMs) presents significant privacy risks. These challenges are particularly pronounced for non-English languages like Japanese, where specialized medical corpora are scarce. While synthetic data generation offers a potential privacy-preserving alternative, its effectiveness in capturing complex clinical nuances—such as negation and contextual dependencies—to train robust classification models without any real-world training data has not been fully established.

**Objective:**

This study aimed to develop a context-aware sentence classification model for Japanese radiology reports using an entirely synthetic training pipeline, thereby eliminating reliance on real-world clinical data during the development phase. Furthermore, we sought to evaluate the generalizability of this approach by validating the model’s performance on diverse, multi-institutional, real-world reports.

**Methods:**

Japanese radiology reports (n=3104) were generated using GPT-4.1 and automatically annotated at the sentence level into 4 categories (background, positive finding, negative finding, and continuation) using GPT-4.1-mini. The synthetic data were partitioned into training (n=2670), validation (n=334), and test (n=100) sets. We fine-tuned several models, including lightweight local LLMs (Qwen3 and Llama 3.2 series) using low-rank adaptation and Japanese text classification models (Bidirectional Encoder Representations from Transformers [BERT]-base Japanese v3, Japanese Medical Robustly Optimized BERT Pretraining Approach [JMedRoBERTa]-base, and ModernBERT-Ja-130M). External validation was performed using 280 real-world reports (3477 sentences) from 7 institutions in the Japan Medical Image Database, with ground-truth labels established by board-certified radiologists. Evaluation metrics included accuracy, macro-averaged *F*_1_ (macro *F*_1_) score, and positive predictive value for positive findings (PPV_1).

**Results:**

All models achieved high performance on the synthetic test set (accuracy: 0.938‐0.951; macro *F*_1_-score: 0.924‐0.940). Overall performance declined on the external validation dataset (accuracy: 0.783‐0.813; macro *F*_1_-score: 0.761‐0.790), reflecting distributional differences between synthetic and real-world reports; however, PPV_1 remained stable and high across datasets (eg, 0.957 on the synthetic test set vs 0.952 on the external validation dataset for Qwen3 [4B]). Parsing errors occurred in LLM-based approaches (19‐260 sentences, 0.55%‐7.48% in the external dataset).

**Conclusions:**

This study demonstrates the feasibility of developing context-aware sentence classification models for Japanese radiology reports using a training pipeline based entirely on synthetic data. The stability of PPV_1 indicates that the models successfully captured the essential clinical terminology and linguistic patterns required to identify positive findings in real-world reports, despite the observed performance degradation during external validation. This approach substantially reduces manual annotation requirements and privacy risks, providing a scalable foundation for constructing structured radiology datasets to support the development of clinically relevant medical artificial intelligence models.

## Introduction

In recent years, vision-language models (VLMs) have been increasingly applied to medical image analysis [1,2]. VLMs are multimodal architectures that commonly integrate large language models (LLMs) for textual processing with visual encoders for image representation, enabling tasks such as image captioning, visual question answering, and image-text matching [3]. In the medical domain, VLMs are typically trained on paired image-text data to associate imaging findings with their corresponding textual descriptions, supporting applications including automated report generation, cross-modal retrieval, and clinical decision support [4-6]. Training these models requires large-scale image-text pairs, and both the quality and quantity of such pairs critically affect the performance of the model [7,8].

Several large-scale, English-language medical image-text datasets, such as the Medical Information Mart for Intensive Care Chest X-Ray (MIMIC-CXR) and CT-RATE, have recently been released [9-11]. These resources have become foundational for training VLMs. Consequently, many state-of-the-art VLMs are predominantly trained and evaluated on English data, largely due to the sheer volume of available resources. When applied to other languages, these models often exhibit performance degradation due to linguistic disparities, such as variations in negation, uncertainty expressions, and reporting conventions across institutions [12]. This is especially true for languages such as Japanese; while they are well-supported in general-domain natural language processing (NLP), the scarcity of public radiology-specific corpora creates a low-resource environment in the medical domain [13,14]. Although some non-English resources such as PadChest exist, they remain limited in scale or scope [15,16]. Simple cross-lingual transfer or translation-based approaches may fail to capture language-specific clinical nuances and local disease prevalence. Furthermore, many publicly available datasets are biased toward prototypical or specific disease cases and do not adequately reflect the diversity of findings and contexts encountered in daily clinical practice [17].

To develop VLMs that can be used in real-world radiology workflows across different countries, it is essential to establish and continuously curate language-specific datasets that reflect local clinical practices. However, radiology reports are typically unstructured free text, exhibiting significant variations in structure and style across institutions and radiologists. They often contain diverse sentence types, including background information, positive findings, negative findings, and continuation from previous sentences. Consequently, simple sentence segmentation is inadequate because sentence boundaries often do not align with thematic transitions, and reports frequently include content that is irrelevant to the actual image findings. Therefore, constructing automated image-text pairs requires text processing—specifically, context-aware sentence classification—as a preliminary step before alignment. While commercial LLMs have shown success in radiology tasks, developing a pipeline that transmits clinical reports to external services is often unacceptable due to ethical, regulatory, and privacy concerns [13,18,19]. Beyond these, economic and technical factors pose substantial challenges; high cumulative application programming interface costs and nontransparent model updates hinder large-scale data curation and compromise reproducibility [20,21]. Consequently, local models are necessary; however, manual annotation for in-house training data is extremely labor-intensive [22,23].

To address these challenges, we leveraged a commercial LLM solely to generate synthetic Japanese radiology reports and perform automated sentence-level annotation, eliminating the need for labor-intensive manual annotation. This synthetic dataset was used to fine-tune lightweight local LLMs, enabling efficient model development while strictly safeguarding patient privacy by avoiding the use of actual clinical data. This study aimed to develop a context-aware sentence classification model for segmenting Japanese radiology reports into finding-level textual units that are suitable for pairing with images for VLM training. We investigated the feasibility of executing the entire training pipeline exclusively using synthetic data, with validation performed on multi-institutional, real-world datasets.

## Methods

### Ethical Considerations

This retrospective human-subject study was approved by the institutional review board of Jichi Medical University Hospital (approval J24-017; approval date: June 10, 2024). The requirement for informed consent was waived by the institutional review board because this study involved a secondary analysis of existing clinical data. All data were anonymized or deidentified prior to analysis in accordance with institutional data governance policies, and no direct personal identifiers were accessible to the research team at any point during the study. No compensation was provided to participants. All figures and images included in this paper were nonidentifiable, and no additional consent for image publication was required.

### Data Synthesis and Annotation (Training Data)

**Figure 1.**
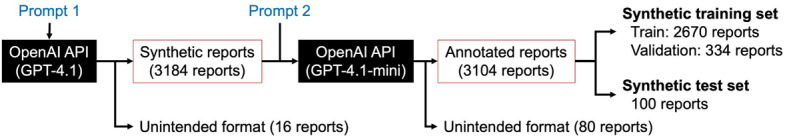
Flowchart of synthetic data generation. Prompt 1 was used for report generation (Section A in Multimedia Appendix 1), and Prompt 2 (system prompt) was used for report annotation (Section B in Multimedia Appendix 1). Synthetic radiology reports were generated using the OpenAI API (GPT-4.1), and these were annotated using the OpenAI API (GPT-4.1-mini). Reports were excluded based solely on structural validity (eg, parsing errors) without semantic filtering. The final dataset was partitioned into training (n=2670), validation (n=334), and test (n=100) sets. API: application programming interface.

We generated synthetic Japanese radiology reports using the OpenAI application programming interface and automatically annotated each sentence into 4 predefined categories (Figure 1). Initially, we attempted to generate 3200 reports using GPT-4.1. To ensure the generation of diverse reports, we explicitly defined various attributes and their candidate values—including radiologist experience level, reporting style and structural preferences, patient demographics, anatomical site, reason for examination, image quality and coverage scope, presence of prior examinations, disease category, diagnostic certainty, and disease rarity—and randomly combined and selected them for each report (Section A in Multimedia Appendix 1).

Subsequently, these reports were automatically annotated using GPT-4.1-mini, where sentences were assigned to one of the following 4 categories: label 0 (background) for nonfinding information within the findings section, including clinical history, prior study comparisons, technical assessments, and demographics; label 1 (positive finding) to denote the presence of abnormalities; label 2 (negative finding) to denote the absence of abnormalities, with label 1 taking precedence if both coexist in a single sentence; and label 3 (continuation) for sentences that provide supplementary details to the preceding sentence without constituting a standalone finding. Representative examples for each label are provided in Table 1, and the system prompt used for automated annotation is detailed in Section B in Multimedia Appendix 1.

After excluding reports with unintended formats, including parsing errors (failure to produce a valid JSON structure), the final dataset comprised 2670 training data, 334 validation data, and 100 test reports. For the synthetic test set, sentence-level annotation into the 4 predefined categories was manually performed by 2 board-certified radiologists, and a consensus was reached for each sentence.

**Table 1.** Definitions of sentence labels and examples.

Label	Definitions	Examples
0=Background	Background information not related to current imaging findings	“Patient has a history of right lung cancer surgery.” “Comparison with previous CT^a^ from October 2024.”
1=Positive finding	Description of a distinct positive finding	“A 2-cm hypervascular lesion is observed in Segment 8 of the liver.” ”Mild pleural effusion is present on the right side.”
2=Negative finding	Description of a distinct negative finding (absence of abnormalities)	“No significant lymphadenopathy is noted.” “There are no signs of intracranial hemorrhage.”
3=Continuation	Sentences that modify or describe the preceding finding and do not stand alone.	“It shows gradual washout on the delayed phase.” “This lesion appears stable compared to the previous exam.”

a CT: computed tomography.

### External Validation Dataset

To evaluate whether models trained solely on synthetic data generalize to real-world clinical practice, we constructed a multi-institutional external validation dataset using reports from the Japan Medical Image Database (J-MID) [24], a nationwide repository of real-world radiological images and reports collected from multiple Japanese institutions. Instead of selecting curated or disease-specific cases, we adopted a snapshot sampling strategy to reflect routine clinical practice. Specifically, we randomly sampled 40 computed tomography reports from each of the 7 participating institutions within the J-MID, resulting in a total of 280 reports dated October 1, 2024. The dataset demonstrated varying imaging coverage—torso (n=141, 50.4%), chest (n=64, 22.9%), abdomen/pelvis (n=20, 7.1%), head (n=19, 6.8%), whole body (n=12, 4.3%), neck (n=8, 2.9%), and others (n=16, 5.7%)—including both noncontrast (n=176, 62.9%) and contrast-enhanced (n=104, 37.1%) studies. For text preprocessing, reports were segmented into sentences using line breaks and periods (“.”). Sentence-level annotation into the 4 predefined categories was manually performed by 2 board-certified radiologists, and a consensus was reached for each sentence.

### Model Selection

We used both LLMs and Japanese text classification models to construct and compare sentence classification models. To ensure that the training could be handled on a single NVIDIA RTX6000 Ada graphics processing unit (GPU) (48 GB memory), we limited the model size to approximately 4 billion parameters (4B). For LLMs, we fine-tuned Qwen3 (0.6B, 1.7B, 4B) and Llama 3.2 (1B, 3B) using low-rank adaptation (LoRA) [25,26]. For text classification, we selected 3 Japanese models based on the BERT (Bidirectional Encoder Representations from Transformers), RoBERTa (Robustly Optimized BERT Pretraining Approach), and ModernBERT architectures [27-29]. First, BERT base Japanese v3 was included as a widely used model in Japanese general and medical NLP tasks, including radiology-focused applications [30,31]. Second, JMedRoBERTa [Japanese Medical RoBERTa]-base was utilized because this model is pretrained on large-scale Japanese medical literature [32]. Third, ModernBERT-Ja-130M was selected as a recently proposed architecture that achieves high performance while maintaining strong computational efficiency [33]. Links to the model cards for all models used in this study are provided in Section C in Multimedia Appendix 1.

### Model Training and Inference

To fine-tune the LLM, we adopted the LoRA framework [34]. The configuration was as follows: rank (r)=16, LoRA *α*=32, dropout=0.05, and target modules={q_proj, k_proj, v_proj, o_proj, gate_proj, up_proj, down_proj}. Training was performed for 3 epochs with a batch size of 2 and gradient accumulation of 8 (effective batch size=16). The learning rate was set to 1×10⁻⁴ with a cosine scheduler, including 100 warm-up steps. The maximum sequence length was 2048 tokens. We applied 4-bit quantization using quantized LoRA (QLoRA) with 16-bit mixed-precision for memory efficiency [35]. In this setting, the entire text was provided as input, and the model was trained for a text-generation task to produce structured outputs in JSON format, utilizing the system prompt detailed in Section B in Multimedia Appendix 1. The outputs were subsequently parsed into sentence-level predictions.

For the text classification models, fine-tuning was performed using the HuggingFace Trainer with a maximum sequence length of 512 tokens, a batch size of 16, a learning rate of 2×10⁻⁵, and 3 training epochs. Mixed precision training with 16-bit mixed precision was applied, and the best checkpoint was selected according to the macro-averaged *F*_1_ (macro *F*_1_) score of the validation set. To incorporate contextual information, we used a sentence-centered local context window consisting of the target sentence and its 2 preceding and 2 following sentences. This approach ensures the inclusion of sufficient local context while remaining within the 512-token limit typical of BERT-based models. During inference, models were applied to individual reports in a sentence-wise manner using the same context window configuration as in training.

### Evaluation Metrics

To provide a rigorous assessment of both the automated annotations and the fine-tuned models, we defined the following standard classification metrics. Classification accuracy was calculated as the ratio of correctly predicted labels to the total number of sentences.

.$$Accuracy\;=\;\frac{Number\;of\;correct\;predictions}{Total\;number\;of\;sentences}$$

To ensure a balanced evaluation under class imbalance, we additionally adopted the macro *F*_1_-score. For each class i(i∈{0,1,2,3}) (0=background, 1=positive finding, 2=negative finding, and 3=continuation), precision $(P_{i})$, recall $\left(R_{i}\right)$, and *F*_1_-score $\left({F1}_{i}\right)$ were defined as follows:


$$P_{i}\;=\frac{TP_{i}}{TP_{i}\;+\;FP_{i}}\;,R_{i}\;=\frac{TP_{i}}{TP_{i}\;+\;FN_{i}}\;,\;F1_{i}\;=\frac{2\;\times \;P_{i}\;\times \;R_{i}}{P_{i}\;+\;R_{i}}$$

The macro *F*_1_-score was calculated as the arithmetic mean of these class-specific scores:


$$MacroF1=\frac{1}{4}\sum_{i=0}^{3}{F1}_{i}$$

In addition, we reported the positive predictive value for label 1 (PPV_1), corresponding to $P_{1}$ in this framework. This metric was included because label 1 represents positive imaging findings, and high precision for this class is essential for ensuring the reliability of extracted image-text pairs. We also reported the recall for label 1 (Recall_1) and class-specific *F*_1_-score for label 1 (F1_1) to evaluate dataset coverage and the overall precision-recall balance, corresponding to $R_{1}$ and F1_1, respectively, in the above framework.

First, to validate the quality of the automated sentence-level annotations generated by GPT-4.1-mini, we compared them against the radiologist consensus on the synthetic test set. Agreement was quantified using classification accuracy, macro *F*_1_-score, and Cohen κ coefficient. Second, for the LLM outputs, we quantified the incidence of parsing errors and aggregated these error rates by facility. Third, the performance of all models was evaluated on both the synthetic test set and external validation datasets using accuracy, macro *F*_1_-score, PPV_1, Recall_1, and F1_1. For LLM-based approaches, sentences associated with parsing errors were excluded from subsequent performance analyses. Fourth, we performed a targeted error analysis. We reviewed 40 reports from the institution with the highest parsing error rate to identify recurring patterns. Additionally, we examined the best-performing model’s errors by extracting false-positive label 1 predictions to identify the primary reasons for these misclassifications.

## Results

Table 2 summarizes the distribution of labels in the datasets used in this study. In the synthetic dataset, label 2 was the most frequent category at 44%, whereas label 1 predominated in the external validation dataset at 42.6%. Synthetic reports exhibited higher verbosity with a mean (SD) character count of 359.1 (SD 138.9) compared to 224.4 (SD 125.9) in the external dataset, despite having similar mean sentence counts (11.2, SD 4.0 vs 12.4, SD 6.3). Substantial variability was observed across external institutions.

**Figure 2.**
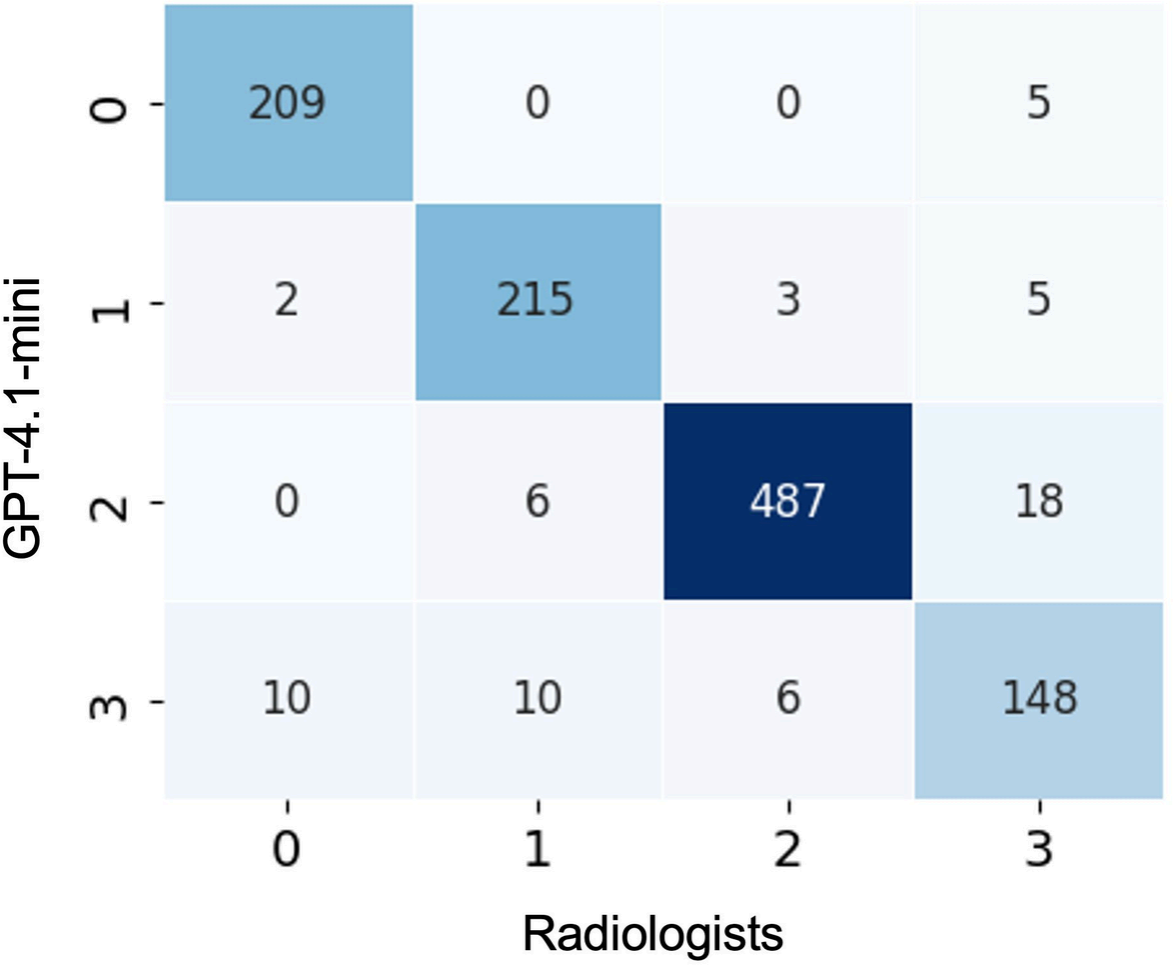
Confusion matrix of GPT-4.1-mini versus radiologist consensus on the synthetic test set. Labels represent the following categories: 0=background, 1=positive findings, 2=negative findings, and 3=continuation. Per-class precision/recall: label 0=0.946/0.977; label 1=0.931/0.956; label 2=0.982/0.953; and label 3=0.841/0.851.

Figure 2 presents the confusion matrix for the annotation performance of GPT-4.1-mini compared with the radiologist consensus on the synthetic test set. The classification accuracy, macro *F*_1_-score, and Cohen κ were 0.942, 0.929, and 0.917, respectively.

**Table 2.** Distribution of the dataset.

Dataset	Label 0^a^, n (%)	Label 1^a^, n (%)	Label 2^a^, n (%)	Label 3^a^, n (%)	Characters/report, mean (SD)	Sentences/report, mean (SD)
Synthetic dataset (3104 reports)						
Total	6593 (18.9)	7673 (22.0)	15,327 (44.0)	5257 (15.1)	359.1 (138.9)	11.2 (4.0)
Train	5680 (18.9)	6620 (22.1)	13,187 (44.0)	4503 (15.0)	359.7 (138.5)	11.2 (4.0)
Validation	699 (18.7)	828 (22.2)	1629 (43.6)	580 (15.5)	360.1 (141.6)	11.2 (3.9)
Test	214 (19.0)	225 (20.0)	511 (45.5)	174 (15.5)	338.6 (139.2)	11.2 (4.3)
External validation dataset (280 reports)						
Total	531 (15.3)	1480 (42.6)	1001 (28.8)	465 (13.4)	224.4 (125.9)	12.4 (6.3)
Institution A	64 (11.6)	233 (42.2)	196 (35.5)	59 (10.7)	304.7 (127.4)	13.8 (6.1)
Institution B	68 (13.9)	200 (40.9)	182 (37.2)	39 (8.0)	202.5 (73.2)	12.2 (4.4)
Institution C	58 (20.4)	122 (42.8)	58 (20.4)	47 (16.5)	148.3 (87.1)	7.1 (3.7)
Institution D	77 (18.3)	150 (35.6)	132 (31.4)	62 (14.7)	135.2 (70.0)	10.5 (5.3)
Institution E	75 (15.5)	201 (41.5)	166 (34.3)	42 (8.7)	186.6 (81.8)	12.1 (4.9)
Institution F	89 (12.4)	337 (47.1)	181 (25.3)	108 (15.1)	339.3 (138.3)	17.9 (7.6)
Institution G	100 (18.8)	237 (44.6)	86 (16.2)	108 (20.3)	254.0 (128.2)	13.3 (6.3)

a Labels represent the following sentence categories: 0=background, 1=positive findings, 2=negative findings, and 3=continuation. Values in parentheses represent percentages of the row total.

Table 3 presents the performance of the models on the internal test set, which consisted of 1124 sentences. Parsing errors were observed only in Qwen3 (0.6B) (3/1124 sentences, 0.27%), and no errors were observed in the other models. Accuracy ranged from 0.938 to 0.951; macro *F*_1_-scores ranged from 0.924 to 0.940; PPV_1 ranged from 0.927 to 0.957; Recall_1 ranged from 0.898 to 0.960; and F1_1 ranged from 0.921 to 0.951.

Table 4 shows the results on the external validation dataset, which comprised 3477 sentences from 280 reports. Parsing errors were observed only in the LLM group, ranging from 19 to 260 sentences (0.55%‐7.48%), whereas no errors were observed in the text classification models. Accuracy ranged from 0.783 to 0.813; macro *F*_1_-score ranged from 0.761 to 0.790; PPV_1 ranged from 0.897 to 0.952; Recall_1 ranged from 0.699 to 0.733; and F1_1 ranged from 0.794 to 0.821. Among all the models, Qwen3 (4B) demonstrated the best overall performance, maintaining high accuracy, Macro *F*_1_-score, and PPV_1.

**Table 3.** Results on the internal synthetic test set^a^.

Model	Parsing errors^b^ (n, % of sentences)	Accuracy (95% CI)	Macro *F*_1_^c^ (95% CI)	PPV^d^ for label 1 (95% CI)	Recall for label 1 (95% CI)	F1 for label 1 (95% CI)
LLMs^e^ (name, parameters)						
Qwen3 (0.6B)	3 (0.27)	0.942 (0.930‐0.955)	0.929 (0.913‐0.944)	0.954 (0.926‐0.978)	0.906 (0.870‐0.943)	0.929 (0.906‐0.951)
Qwen3 (1.7B)	0 (0)	0.951 (0.937‐0.963)	0.940 (0.923‐0.955)	0.941 (0.910‐0.970)	0.915 (0.873‐0.947)	0.928 (0.904‐0.948)
Qwen3 (4B)	0 (0)	0.950 (0.937‐0.962)	0.939 (0.922‐0.954)	0.957 (0.930‐0.981)	0.898 (0.860‐0.941)	0.927 (0.903‐0.950)
Llama 3.2 (1B)	0 (0)	0.949 (0.938‐0.961)	0.940 (0.926‐0.955)	0.949 (0.923‐0.976)	0.911 (0.871‐0.949)	0.930 (0.908‐0.952)
Llama 3.2 (3B)	0 (0)	0.942 (0.928‐0.956)	0.927 (0.908‐0.944)	0.927 (0.889‐0.960)	0.915 (0.884‐0.948)	0.921 (0.897‐0.945)
Text classification models						
BERT^f^ base Japanese v3	0 (0)	0.944 (0.931‐0.956)	0.931 (0.914‐0.946)	0.939 (0.907‐0.969)	0.960 (0.931‐0.986)	0.949 (0.930‐0.968)
JMedRoBERTa^g^-base	0 (0)	0.938 (0.924‐0.952)	0.924 (0.905‐0.940)	0.941 (0.910‐0.969)	0.925 (0.886‐0.959)	0.933 (0.909‐0.956)
ModernBERT-Ja-130M	0 (0)	0.948 (0.936‐0.959)	0.933 (0.917‐0.948)	0.951 (0.925‐0.973)	0.951 (0.921‐0.977)	0.951 (0.934‐0.968)

a 95% CIs were estimated using the bootstrap method.

b Parsing errors: failures in JSON parsing during output generation.

c Macro *F*_1_: macro-averaged *F*_1_.

d PPV: positive predictive value.

e LLM: large language model.

f BERT: Bidirectional Encoder Representations from Transformers.

g JMedRoBERTa: Japanese Medical Robustly Optimized BERT Pretraining Approach.

**Table 4.** Results on the external validation dataset^a^.

Model	Parsing errors^b^ (n, % of sentences)	Accuracy (95% CI)	Macro *F*_1_^c^ (95% CI)	PPV^d^ for label 1 (95% CI)	Recall for label 1 (95% CI)	F1 for label 1 (95% CI)
LLMs^e^ (name, parameters)						
Qwen3 (0.6B)	81 (2.33)	0.783 (0.767‐0.802)	0.761 (0.743‐0.780)	0.919 (0.899‐0.937)	0.699 (0.671‐0.725)	0.794 (0.774‐0.813)
Qwen3 (1.7B)	121 (3.48)	0.797 (0.780‐0.815)	0.776 (0.759‐0.794)	0.932 (0.916‐0.948)	0.701 (0.673‐0.729)	0.800 (0.779‐0.822)
Qwen3 (4B)	19 (0.55)	0.813 (0.796‐0.829)	0.790 (0.771‐0.808)	0.952 (0.939‐0.964)	0.721 (0.695‐0.746)	0.821 (0.802‐0.839)
Llama 3.2 (1B)	260 (7.48)	0.797 (0.779‐0.813)	0.773 (0.754‐0.790)	0.916 (0.897‐0.933)	0.724 (0.697‐0.751)	0.808 (0.789‐0.829)
Llama 3.2 (3B)	70 (2.01)	0.806 (0.788‐0.822)	0.782 (0.763‐0.800)	0.919 (0.901‐0.935)	0.733 (0.707‐0.759)	0.816 (0.797‐0.834)
Text classification models						
BERT^f^ base Japanese v3	0 (0)	0.788 (0.771‐0.804)	0.770 (0.752‐0.786)	0.897 (0.877‐0.916)	0.716 (0.688‐0.743)	0.796 (0.774‐0.816)
JMedRoBERTa^g^-base	0 (0)	0.799 (0.781‐0.815)	0.783 (0.765‐0.799)	0.932 (0.915‐0.948)	0.709 (0.680‐0.737)	0.806 (0.786‐0.826)
ModernBERT-Ja-130M	0 (0)	0.801 (0.784‐0.818)	0.782 (0.764‐0.799)	0.917 (0.898‐0.934)	0.722 (0.697‐0.748)	0.808 (0.790‐0.827)

a 95% CIs were estimated using the bootstrap method.

b Parsing errors: failures in JSON parsing during output generation.

c Macro *F*_1_: macro-averaged *F*_1_.

d PPV: positive predictive value.

e LLM: large language model.

f BERT: bidirectional encoder representations from transformers.

g JMedRoBERTa: Japanese Medical Robustly Optimized BERT Pretraining Approach.

Table 5 summarizes the accuracy and sentence-level parsing errors for each institution. In institution C, which exhibited a notably high incidence of parsing errors, a detailed review of 40 reports revealed that all reports with failed parsing contained specific notations such as “[#1]” or “[#1‐2]” within the text. These markers appeared to be used for referencing attached images, potentially reflecting a reporting style specific to that institution or individual radiologists.

**Figure 3.**
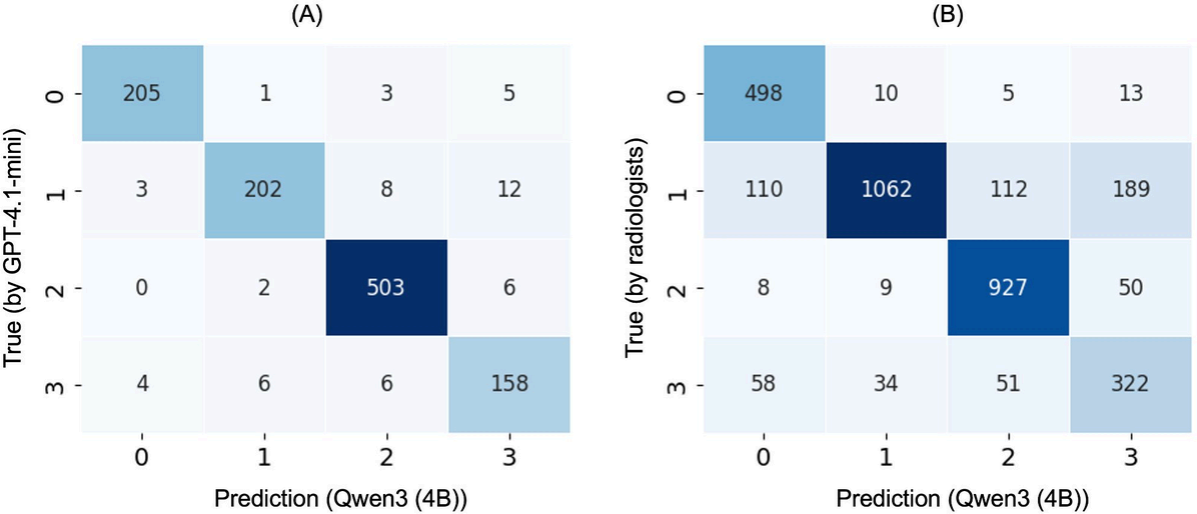
Performance of Qwen3 (4B) on the (A) internal synthetic test set and (B) external validation datasets. Labels represent the following categories: 0=background, 1=positive findings, 2=negative findings, and 3=continuation. Per-class precision/recall on the synthetic test set: Label 0 = 0.967/0.958; Label 1 = 0.957/0.898; Label 2 = 0.967/0.984; Label 3 = 0.873/0.908. On the external validation dataset: Label 0 = 0.739/0.947; Label 1 = 0.952/0.721; Label 2 = 0.847/0.933; and Label 3 = 0.561/0.692.

Figure 3 shows the confusion matrices of Qwen3 (4B), the top-performing model, for the synthetic test set and external validation datasets.

An error analysis was conducted on the predictions of Qwen3 (4B) to identify factors contributing to the degradation of PPV_1. Among sentences incorrectly predicted as label 1, 3 recurring patterns were observed: (1) for label 0, 80% (8/10) of errors involved mistaking patient status or treatment history for active findings; (2) for label 2, 44% (4/9) of errors occurred in sentences mentioning a lesion name with weak negation or stability descriptors (eg, “unclear” or “maintained reduction”); and (3) for label 3, 41% (14/34) of misclassifications involved sentences describing differential diagnoses related to findings in the preceding text.

**Table 5.** Accuracy and parsing errors by institution^a^.

Model/institution	A	B	C	D	E	F	G
LLMs^b^ (name, parameters)							
Qwen3 (0.6B)	0.835 (1)	0.807 (3)	0.737 (2)	0.810 (1)	0.766 (4)	0.806 (9)	0.696
Qwen3 (1.7B)	0.842 (2)	0.797 (1)	0.746 (8)	0.858 (2)	0.786	0.814 (5)	0.724
Qwen3 (4B)	0.855	*0.816*	*0.754* (1)	*0.884* (1)	*0.805*	0.817 (1)	0.742
Llama 3.2 (1B)	0.853 (3)	0.810 (5)	0.722 (11)	0.835 (5)	0.805 (4)	0.806 (8)	0.719
Llama 3.2 (3B)	*0.857*	0.808 (6)	0.720 (6)	0.867 (1)	0.786 (1)	*0.826* (4)	0.734
Text classification models							
BERT^c^ base Japanese v3	0.844	0.81	0.712	0.77	0.783	0.813	0.74
JMedRoBERTa^d^-base	0.844	0.814	0.74	0.796	0.793	0.815	*0.755*
ModernBERT-Ja-130M	0.841	0.802	0.709	0.834	0.8	0.822	0.753

a Numbers indicate accuracy; numbers in parentheses indicate the number of report-level parsing errors. Italicized values indicate the highest accuracy for each institution.

b LLM: large language model.

c BERT: Bidirectional Encoder Representations from Transformers.

d JMedRoBERTa: Japanese Medical Robustly Optimized BERT Pretraining Approach.

## Discussion

### Principal Findings

In this study, we developed context-aware sentence classification models for Japanese radiology reports using synthetic data and evaluated the models on multi-institutional radiology reports. All models demonstrated high performance on the internal synthetic test set (accuracy: 0.938‐0.951; macro *F*_1_-score: 0.924‐0.940). Although performance declined on the external validation dataset (accuracy: 0.783‐0.813; macro *F*_1_-score: 0.761‐0.790), PPV_1 remained stable and comparable to the internal results (eg, 0.957 vs 0.952 for Qwen3 [4B]). This discrepancy suggests that while the distributional gap between synthetic and real-world reports posed challenges for overall classification, the models effectively captured the core linguistic patterns and medical terminology associated with positive findings. From a practical standpoint, the stability of PPV_1 is particularly important, as it indicates that models trained solely on synthetic data can reliably identify findings from diverse clinical reports without a high rate of false positives. This robustness supports the quality of the resulting image-text pairs, which is a prerequisite for the efficient large-scale development of VLMs. Although PPV_1 remained stable across datasets, Recall_1 declined from 0.898 to 0.960 on the synthetic test set to 0.699 to 0.733 on the external validation dataset, indicating that approximately 27% to 30% of true positive findings were not captured. In the context of VLM dataset construction, this precision-recall balance has practical implications. False-positive image-text pairs, where a nonfinding sentence is incorrectly paired with an image, can actively introduce noise into model training. In contrast, false negatives reduce dataset coverage but do not compromise the quality of the retained pairs. Therefore, the observed high-precision, moderate-recall profile is considered to be acceptable for the initial construction of reliable image-text datasets, while improving recall through prompt refinement and domain adaptation remains an important direction for future work.

### Prior Work and the Sentence-Level Approach

Previous studies in radiology report that structuring has predominantly utilized entity-level extraction (eg, extracting specific disease names) or graph-based representations involving nodes and edges [36-38]. These paradigms offer distinct advantages: entity-level extraction allows for the highly granular identification of specific clinical findings and facilitates the precise handling of negation and uncertainty expressions, while graph-based approaches excel at representing complex relationships between anatomical locations and pathologies. More recently, NLP applications have expanded to include the labeling of anatomical phrases, paraphrasing of clinical statements, and full report structuring [37,39,40].

In contrast, this study adopted a sentence-level labeling approach. Although this method faces challenges with sentences containing multiple distinct findings (eg, “A liver cyst is present, but no renal cyst is observed”) [41], our policy prioritized label 1. In many clinical instances, positive and negative statements coexisting in a single sentence refer to the same lesion (eg, “A hypodense lesion is present in the liver, but it shows no corresponding contrast enhancement”). Our framework categorizes such descriptions as label 1. If the clauses in this example were split into 2 separate sentences, they would be labeled as label 1 followed by label 3. By utilizing the combination of label 1 and its associated label 3 in downstream tasks, nearly equivalent semantic information can be obtained regardless of whether the description is contained within a single sentence or distributed across multiple sentences. Therefore, we argue that this approach remains robust for practical clinical applications. As a preliminary analysis, we examined the potential impact of multiclause sentences on the classification performance using Qwen3 (4B) predictions on the external validation dataset; the results are detailed in Section F in Multimedia Appendix 1. While implementing an LLM-based preprocessing step to segment complex sentences into distinct, meaning-preserving statements could further refine this pipeline, we deferred this in this study to avoid increasing procedural complexity and the difficulty of establishing evaluation metrics.

More importantly, we believe that preserving sentence-level expressions—rather than reducing them to simple entity extraction—is highly beneficial for VLM training. This approach retains the nuanced phrasing and the radiologist’s diagnostic thought process. Preserving these linguistic characteristics provides richer textual information for the model to learn the association between visual findings and professional reporting styles, which is essential for developing clinically relevant models [4,42].

### Challenges in Clinical Diversity and Prompt Optimization

The variability in performance across institutions (Table 5) provides key insights for implementation. Table 2 reveals significant diversity in report length and label distribution. Notably, facilities with metrics similar to the synthetic data—such as report length and finding proportions—did not always yield the highest accuracy. This suggests that clinical diversity involves complex factors beyond aggregate metrics, including disease prevalence, institution-specific rules, and individual radiologist styles. Although our prompts incorporated various elements, they do not yet fully align with real-world practice. Potential refinements include adjusting demographic granularity (eg, using numerical values instead of categorical descriptors), reweighting clinical scenarios (eg, disease frequency, emergency vs inpatient settings, or the prevalence of poor image quality and motion artifacts), and fine-tuning class distributions (eg, the ratio of positive to negative findings). Future research must refine prompt design to address this reporting diversity, which remains the primary challenge in utilizing a purely synthetic training set.

Error analyses identified several technical and clinical challenges. Parsing errors in institution C likely resulted from image-referencing notations such as “[#1],” which the LLM may have misinterpreted as structural control characters. For Qwen3 (4B), false-positive label 1 predictions revealed specific patterns: (1) 80% (8/10) of label 0 errors involved mistaking patient history for active findings; (2) 44% (4/9) of label 2 errors involved lesion names paired with weak negations or stability descriptors, such as “unclear” or “maintained reduction”; and (3) 41% (14/34) of label 3 errors occurred in sentences describing differential diagnoses related to preceding findings. These results emphasize the importance of incorporating complex negations, facility-specific symbols, and sophisticated clinical contexts into future synthetic data prompts [43,44]. While this study focused on validating general extraction performance, institutional domain adaptation and post hoc prompt adjustments remain subjects for future investigation.

### Practical Considerations for Clinical Deployment

Text classification models, including BERT base Japanese v3, JMedRoBERTa-base, and ModernBERT-Ja-130M, showed stable performance compared to those of small-scale LLMs. From a practical standpoint, these classification models offer distinct advantages in reliability and processing speed (detailed in Section D in Multimedia Appendix 1). Their architecture inherently eliminates the risk of format instabilities while ensuring high throughput even on standard hardware [45]. While requiring more computational resources than BERT-based models, the decision to evaluate small-scale LLMs was similarly rooted in the need for on-premise feasibility. These models with under 4 billion parameters can operate on a single mid-range GPU with 12 to 24 GB of Video RAM (eg, NVIDIA RTX 4090 or RTX 4070 Ti Super). This allows institutions to maintain strict data privacy and ensure operational stability without relying on external cloud-based services.

Parsing errors remained a unique challenge to the LLM group [46]. While we considered using vLLM, an open-source library optimized for high-throughput LLM serving, to implement JSON mode, we intentionally opted against it. JSON mode is a constrained decoding technique provided by the inference framework rather than a native capability of the LLMs themselves. Our primary objective was to evaluate the models’ inherent ability to follow structural instructions without external enforcement. Furthermore, we found that such rigid constraints could lead to unintended text alterations or premature output truncation. We also investigated whether a re-inference strategy could resolve these parsing errors, with the results detailed in Section E in Multimedia Appendix 1.

### Limitations

This study had some limitations. First, most training labels in the synthetic data were generated automatically without individual human verification. While a quality assessment of the synthetic test set, comprising 1124 sentences from 100 reports, showed high reliability (κ=0.917), the remaining unverified training data may contain annotation noise that potentially constrained the models’ final performance. The iterative refinement of these labels through expert review and subsequent fine-tuning could represent a promising avenue to further enhance model accuracy and robustness.

Second, although Japanese served as a representative example of a low-resource language in the medical domain, the generalizability of this approach to other underrepresented languages has not yet been verified. As noted in the section “Challenges in Clinical Diversity and Prompt Optimization,” there remains room for improvement in our synthetic data generation process. Applying this methodology to other languages would necessitate additional tuning to align with specific linguistic nuances and localized clinical reporting conventions.

Third, the comparison between LLMs and text classification models involved differing input contexts. LLMs processed full reports to leverage their expansive context windows and maximize throughput. In contrast, text classification models utilized a 5-sentence sliding window due to inherent input length constraints. Restricting LLMs to identical local windows would have artificially limited their native processing capacity and practical utility. Therefore, these results should be interpreted as a comparison of each model in its optimal configuration rather than under strictly uniform input conditions.

### Future Directions

Future work should focus on integrating expert-annotated real-world reports for iterative model fine-tuning and establishing a more robust pipeline. This includes the implementation of advanced preprocessing steps, such as LLM-based sentence segmentation, to accurately handle descriptions containing multiple clinical findings. Furthermore, a primary objective will be to generate high-quality image-text pairs from these structured reports to facilitate the development of medical vision-language foundation models.

### Conclusions

This study demonstrated the feasibility of developing context-aware sentence classification models for Japanese radiology reports using a training pipeline based entirely on synthetic data, thereby substantially reducing the need for labor-intensive manual labeling. Across multiple model architectures, high classification performance was achieved on the synthetic test set. Although external validation using multi-institutional, real-world reports showed a consistent pattern of performance degradation, the models maintained a stable positive predictive value for positive findings. These results indicate that the models effectively captured the essential clinical terminology and linguistic patterns required to identify positive imaging findings in real-world reports.

## Supplementary material

10.2196/86365 Multimedia Appendix 1 Prompts for synthetic report generation and annotation, model card information, throughput benchmarks, parsing error analysis, and subanalysis of label 1 predictions by sentence complexity.

## Abbreviations

BERT: Bidirectional Encoder Representations from Transformers
GPU: graphics processing unit
J-MID: Japan Medical Image Database
JMedRoBERTa: Japanese Medical Robustly Optimized BERT Pretraining Approach
LLM: large language model
LoRA: low-rank adaptation
Macro *F*_1_: macro-averaged *F*_1_
MIMIC-CXR: Medical Information Mart for Intensive Care Chest X-Ray
NLP: natural language processing
PPV: positive predictive value
QLoRA: quantized low-rank adaptation
RoBERTa: Robustly Optimized BERT Pretraining Approach
VLM: vision-language model
