# Supplementary material for: Context-Aware Sentence Classification of Radiology Reports Using Synthetic Data: Development and Validation Study
Source: J Med Internet Res. 2026 Apr 10;28:e86365. doi: 10.2196/86365 (PMC13068187; doi:10.2196/86365)
Supplement: Multimedia Appendix 1 [file jmir-v28-e86365-s001.pdf]

# Appendix

## A. Prompts for report generation

### A.1. System prompt

```None
"下記の状況から実際の臨床場面を設定してください。"
"経過や状況、解剖部位、特定の疾患を具体的に設定することがとても重要です。"
"そしてとても具体的で実際にありうる日本語の画像診断レポートを作成してください。"
"所見と診断をJson形式で出力してください。keyは'診断'と'所見'としてください。"
"valueはリスト型としてください。改行する場合は改行コードではなく、リストの次の要素として記入してください。"
```

### A.2. User prompt

*Note: Although the list of anatomical sites largely consists of sex-independent organs, the "pelvic organs" category may encompass sex-specific anatomy. The pipeline does not incorporate a mechanism to actively prevent the generation of reports involving sex-inconsistent anatomical findings.*

```Python
import random

# Parameter pools for prompt generation
experience_levels = ["若手", "中堅", "熟練", "今回の読影分野のプロフェッショナル", "一般的な能力"]
reader_styles = ["簡潔", "丁寧", "できるだけ長い記載","箇条書き形式", "専門用語の多用", "わかりやすさ重視", "(頭部)、(胸部)、(腹部)のように部位ごとに分けての詳細な自然文での記載"]
patient_sexes = ["男性", "女性"]
patient_ages = ["小児", "成人", "高齢者"]
patient_ages_weights = [0.1, 0.5, 0.4]
modalities = ["単純CT", "造影CT", "単純MRI", "造影MRI", "X線", "PET-CT", "PET-CT以外の適切な核医学"]
modalities_weights = [0.3, 0.25, 0.2, 0.15, 0.05, 0.04, 0.01]
sites = ["脾臓", "腎臓", "胆嚢・胆管", "肝臓", "胃", "膵臓", "副腎", "肺", "気管", "甲状腺", "腸管",
         "膀胱", "骨盤臓器", "骨", "心臓", "血管", "関節", "脊髄", "脳"]
quality = ["良好", "適切", "一部で不十分"]
quality_weights = [0.7, 0.299, 0.001]
exam_reasons = [
    "検診異常の精査", "症状の精査・スクリーニング", "術後フォローアップ", "治療効果判定", "外傷後の評価",
    "偶発所見フォロー", "手術前評価"]
disease_categories = [
    "腫瘍性病変", "炎症性・感染性病変", "血管性病変", "先天性・発達異常", "変性疾患", "外傷性病変"]

range_notes = [
    "局所だけでなく、撮像範囲全体の所見を詳細に記載してください",
    "今回の撮像範囲に含まれる他部位の要点も記載してください",
    "該当部分以外の記載は最小限にしてください",
    "全体としてよくかけていますが、まれに誤字・脱字、変換ミスに気づかないことがあります"
]
range_notes_weights = [0.45, 0.30, 0.20, 0.05]

first_lines = [
    "には前回検査があったことを書きましょう",
```

```
    "には実施した検査種の明記、比較した画像検査を記載してください",
    "には実施した検査種を記載してください",
    "には比較した画像検査の有無を記載してください",
]

second_lines = [
    "2行目には臨床情報の要約を記載してください。",
    "2行目には検査の目的を記載してください。",
    ""
]
second_lines_weights = [0.5, 0.1, 0.4]

add_notes = [
    "",
    "その他には気になる所見はありませんでした。",
    "検査目的の他に重要な異常所見をみつけました。",
    "検査目的の他にはあまり重要ではないものの複数の異常所見がありました。",
    "検査目的の他にはあまり重要ではないものの別の異常所見がありました。",
]

lesion_patterns = [
    "{ctg}の1つに特徴的な所見がありました、具体的な疾患を絞り込むことができました",
    "{ctg}の1つと診断できる特徴的な所見がありました",
    "{ctg}を疑う異常所見がったものの、鑑別に挙げるにとどめました",
    "{ctg}を中心に複数の鑑別診断を考慮する異常所見がありましたが、特定の疾患は絞りきれませんでした",
    "common diseaseの1つを第一に考えました",
    "比較的稀ではありますが特定の疾患を考えました",
    "非常に稀な疾患を疑う所見がありました",
    "具体的な疾患名までは絞れませんでしたが、{ctg}の可能性を考慮しました",
    "読影の結果、異常所見はないと考えました",
]

# Prompt template
prompt_template = """
あなたは{reader_experience}の放射線科医で、レポート作成は{reader_style}を好みます。
現在、{patient_age}歳の{patient_sex}の検査を読影しています。
検査理由は{site}の{exam_reason}で{modality}が行われました。検査範囲は適切で、画質は{quality}です。
{lesion_note}。{add_note}
所見の1行目{first_line}。{second_line}
なお、{range_note}。
""".strip()

def generate_lesion_note():
    """Generate a lesion description by selecting a random disease category and pattern."""
    ctg = random.choice(disease_categories)
    pattern = random.choice(lesion_patterns)
    return pattern.format(ctg=ctg)

def generate_prompt():
    """Generate a randomized user prompt for radiology report generation."""
    data = {
        "reader_experience": random.choice(experience_levels),
        "reader_style": random.choice(reader_styles),
        "patient_age": random.choices(patient_ages, weights=patient_ages_weights, k=1)[0],
        "patient_sex": random.choice(patient_sexes),
        "modality": random.choices(modalities, weights=modalities_weights, k=1)[0],
        "quality": random.choices(quality, weights=quality_weights, k=1)[0],
        "site": random.choice(sites),
        "lesion_note": generate_lesion_note(),
```

```
        "exam_reason": random.choice(exam_reasons),
        "disease_category": random.choice(disease_categories),
        "range_note": random.choices(range_notes, weights=range_notes_weights, k=1)[0],
        "first_line": random.choice(first_lines),
        "second_line": random.choices(second_lines, weights=second_lines_weights, k=1)[0],
        "add_note": random.choice(add_notes),
    }

    return prompt_template.format(**data)
```

## A.3. Example of a generated user prompt

```
None
あなたは熟練の放射線科医で、レポート作成は箇条書き形式を好みます。 現在、高齢者歳の男性の検査を読影しています。 検査理由は心臓の偶発所見フォローで単純CTが行われました。検査範囲は適切で、画質は適切です。 非常に稀な疾患を疑う所見がありました。 所見の1行目には比較した画像検査の有無を記載してください。2行目には検査の目的を記載してください。 なお、今回の撮像範囲に含まれる他部位の要点も記載してください。
```

## A.4. Example of a generated report

```
None
{
  "所見": [
    "前回検査画像なし。",
    "検査目的：心臓の偶発所見フォロー。",
    "心臓：左房壁内に限局性の石灰化を伴う結節性病変を認め、稀な心臓腫瘍（心臓骨腫、石灰化肉腫など）が疑われる。",
    "冠動脈：高度狭窄や閉塞を示す明らかな所見なし。",
    "肺野：両側肺に明らかな結節影や浸潤影なし。",
    "縦隔・胸膜：異常リンパ節腫大や胸水貯留なし。",
    "肝臓・上腹部（撮像範囲内）：腫瘤性病変や胆道拡張なし。"
  ],
  "診断": [
    "左房壁内に限局性石灰化を伴う結節性病変。",
    "心臓原発の稀な腫瘍性病変を最も疑う。",
    "鑑別：心臓骨腫、石灰化肉腫、稀な石灰化血栓。",
    "精査目的に心臓MRIまたは心臓超音波検査の追加を推奨。"
  ]
}
```

## B. System Prompt for report annotation

```
None
"""
あなたは放射線画像に特化したテキスト分類モデルです。
入力：キー「finding_lines」に格納された画像所見文のリスト
タスク：各文を順に評価し、以下の基準でラベルを割り当ててください。
0 - 画像所見に関連しない文章（背景情報など）
1 - 文脈中に新たに登場する別個の*陽性*の画像所見を記述した文章。
2 - 文脈中に新たに登場する別個の*陰性*の画像所見を記述した文章。（※１の要素もある場合は１を優先）
3 - この前の文を修飾しているもの、かつ単独では画像所見として成立しない文章（例：前述の病変の性状説明、所見の変化、など）

出力：元の文章をキー、ラベルを値とする JSON オブジェクトのみを返してください。元の文章を改変してはいけません。

例）
入力
finding_lines = [
"比較画像なし。",
"膵体部に径18mm大の境界明瞭な強く造影される内部均一な腫瘤を認めます。",
"腫瘤は動脈相で著明な早期造影効果を示し、門脈相でやや等信号、後期像で等低信号となります。",
"撮像範囲内の他臓器に明らかな異常なし。",
"骨に退行性変化を認めますが、転移はありません。",
]
出力
{
"比較画像なし。": 0,
"膵体部に径18mm大の境界明瞭な強く造影される内部均一な腫瘤を認めます。": 1,
"腫瘤は動脈相で著明な早期造影効果を示し、門脈相でやや等信号、後期像で等低信号となります。": 3,
"周囲への浸潤はありません。": 3,
"撮像範囲内の他臓器に明らかな異常なし。": 2,
"骨に退行性変化を認めますが、転移はありません。": 1
}
```

## C. Model Card Information

All models were accessed via Hugging Face on September 29, 2025

1. Qwen3 (4B) (Qwen/Qwen3-4B).
   Available at: https://huggingface.co/Qwen/Qwen3-4B
2. Llama 3.2 (3B) (meta-llama/Llama-3.2-3B).
   Available at: https://huggingface.co/meta-llama/Llama-3.2-3B
3. BERT base Japanese v3 (tohoku-nlp/bert-base-japanese-v3).
   Available at: https://huggingface.co/tohoku-nlp/bert-base-japanese-v3
4. JMedRoBERTa-base (alabnii/jmedroberta-base-sentencepiece).
   Available at: https://huggingface.co/alabnii/jmedroberta-base-sentencepiece
5. ModernBERT-Ja-130M (sbintuitions/modernbert-ja-130m).
   Available at: https://huggingface.co/sbintuitions/modernbert-ja-130m

## D. Throughput benchmarks across models

We evaluated the throughput of each model for the primary task of report sentence classification using a single NVIDIA RTX 6000 Ada GPU. To ensure a representative measure of processing speed, we selected a single report from the internal test set with a length corresponding to the dataset's average. Throughput was calculated based on 10 repeated inferences, and reported processing times exclude the initial model loading latency.

| | Reports / min | Average time per report (sec) |
|---|---|---|
| **LLMs** | | |
| Qwen3 (0.6B) | 5.48 | 10.94 |
| Qwen3 (1.7B) | 5.22 | 11.48 |
| Qwen3 (4B) | 4.06 | 14.76 |
| Llama 3.2 (1B) | 9.16 | 6.55 |
| Llama 3.2 (3B) | 5.22 | 11.50 |
| **Text Classification Models** | | |
| BERT base Japanese v3 | 1,454.94 | 0.041 |
| JMedRoBERTa-base | 712.69 | 0.084 |
| ModernBERT-Ja-130M | 1,645.23 | 0.036 |

## E. Parsing Errors and Re-inference Results

During the initial inference, the temperature was set to 0.0 to ensure deterministic output. For reports that resulted in a parsing error, defined as a failure to generate a valid JSON structure, we performed re-inference with the temperature increased to 0.5. This increase was intended to introduce stochasticity to facilitate the generation of a valid format, with a maximum of 20 attempts allowed per report. While this re-inference strategy was investigated to assess the recoverability of structural errors, all performance metrics reported in the Results section are based solely on the initial inference attempt.

| Model | Synthetic test cases | | External validation dataset | |
|---|---|---|---|---|
| | Parse errors | Resolved | Parse errors | Resolved |
| Qwen3 (0.6B) | 3 (0.3%) / 1 (1.0%) | 3 / 1 | 81 (2.3%) / 20 (7.1%) | 39 / 7 |
| Qwen3 (1.7B) | 0 | 0 | 121 (3.5%) / 18 (6.4%) | 86 / 12 |
| Qwen3 (4B) | 0 | 0 | 19 (0.5%) / 3 (1.1%) | 18 / 2 |
| Llama 3.2 (1B) | 0 | 0 | 241 (6.9%) / 36 (12.9%) | 86 / 10 |
| Llama 3.2 (3B) | 0 | 0 | 70 (2.0%) / 18 (6.4%) | 47 / 9 |

Values are presented as sentence-level counts (percentage) / case-level counts (percentage).

## F. Sub-analysis of Label 1 predictions by sentence complexity

To assess whether multi-clause sentences affect classification performance, we categorized all ground-truth Label 1 sentences in the external validation dataset by their compositional complexity and examined Qwen3 (4B) predictions. Sentences with parsing errors were excluded.

| Category | Correct | Total | Accuracy |
|---|---|---|---|
| **All Label 1 sentences** | 1,062 | 1,473 | 72.1% |
| **Contains multiple positive findings** | 83 | 122 | 68.0% |
| **Contains negative(Label 2) component** | 6 | 8 | 75.0% |
| **Contains continuation(Label 3) component** | 195 | 291 | 67.0% |

Sentences containing multiple positive findings or continuation components appeared to have slightly lower accuracy compared to the overall dataset. Sentences containing negative components did not appear to show a clear decline, although the small sample size (n=8) limits the interpretability of this finding.
